# Hydrogen alleviates hypoxic–ischaemic brain damage in neonatal rats by inhibiting injury of brain pericytes

**DOI:** 10.1111/jcmm.18505

**Published:** 2024-07-12

**Authors:** Hui Li, Hao Sun, Shiping Li, Lingyi Huang, Mingfu Zhang, Shaopu Wang, Qian Liu, Junjie Ying, Fengyan Zhao, Xiaojuan Su, Dezhi Mu, Yi Qu

**Affiliations:** ^1^ Department of Pediatrics/Key Laboratory of Birth Defects and Related Diseases of Women and Children (Ministry of Education)/NHC Key Laboratory of Chronobiology, West China Second University Hospital Sichuan University Chengdu China; ^2^ Department of Orthodontics, West China College of Stomatology/State Key Laboratory of Oral Diseases Sichuan University Chengdu China

**Keywords:** brain pericytes, cerebrovascular function, hydrogen, hypoxia–ischaemia, neural repair, oxidative stress

## Abstract

Hypoxia‐ischaemia (HI) can induce the death of cerebrovascular constituent cells through oxidative stress. Hydrogen is a powerful antioxidant which can activate the antioxidant system. A hypoxia‐ischaemia brain damage (HIBD) model was established in 7‐day‐old SD rats. Rats were treated with different doses of hydrogen‐rich water (HRW), and brain pericyte oxidative stress damage, cerebrovascular function and brain tissue damage were assessed. Meanwhile, in vitro‐cultured pericytes were subjected to oxygen–glucose deprivation and treated with different concentrations of HRW. Oxidative injury was measured and the molecular mechanism of how HRW alleviated oxidative injury of pericytes was also examined. The results showed that HRW significantly attenuated HI‐induced oxidative stress in the brain pericytes of neonatal rats, partly through the Nrf2‐HO‐1 pathway, further improving cerebrovascular function and reducing brain injury and dysfunction. Furthermore, HRW is superior to a single‐cell death inhibitor for apoptosis, ferroptosis, parthanatos, necroptosis and autophagy and can better inhibit HI‐induced pericyte death. The liver and kidney functions of rats were not affected by present used HRW dose. This study elucidates the role and mechanism of hydrogen in treating HIBD from the perspective of pericytes, providing new theoretical evidence and mechanistic references for the clinical application of hydrogen in neonatal HIE.

## 1 INTRODUCTION

Neonatal hypoxic–ischaemic encephalopathy (HIE) is a refractory neurological disease in the neonatal period that can lead to neonatal death or legacy neurological sequelae.^1^ The pathogenesis of HIE is complex. Currently, the exact mechanism has not been clarified, and there is a lack of specific treatment in clinical practice.^2^ Therefore, it is important to investigate the pathogenesis and treatment of HIE. HIE is associated with multiple cell injury.^3^ In addition to the damage to nerve cells including neurons, astrocytes and oligodendrocytes, cerebral vascular dysfunction caused by hypoxia ischaemia (HI) is an important factor leading to the occurrence and development of brain injury.^4^

Pericytes are the main cells that make up cerebral blood vessels. They are embedded in the basement membrane of cerebral microvascular endothelial cells and play an important role in maintaining the blood–brain barrier (BBB), regulating cerebral blood flow (CBF) and modulating immune cell entry into the central nervous system.^5^ Therefore, pericytes play a key role in the occurrence and development of central nervous system diseases. Under normal physiological conditions, increased neuronal excitability or neurotransmitter release can cause pericytes to relax, leading to capillary dilation. When cerebral ischaemia occurs, many pericytes die, leading to irreversible contraction of capillaries and severe cerebrovascular dysfunction.^6^ Another study found that death and sustained abnormal contraction of pericytes during ischaemia led to a reduction in capillary diameter and the arrangement of red blood cells appeared intermittent, affecting blood flow perfusion.^7^ NO synthase inhibitors and oxygen‐free radical scavengers can reduce pericyte death, thus relieving sustained abnormal contraction of pericytes and restoring microcirculation.^8^, ^9^ Pericyte death inhibition can be expected to become an important way to alleviate cerebrovascular dysfunction after HI.

Cerebral vessels are highly sensitive to oxidative stress. HI induces NO synthase activation in vascular endothelial cells, leading to NO production. Superoxide anions (O2^−^) are also produced during vascular damage. O2^−^ reacts with NO to form the peroxynitrite anion (ONOO^−^), which is a strong inducer of DNA damage that can cause oxidative stress in vascular cells.^10^ Because the number of dead pericytes after HI is much higher than vascular endothelial cells, the study of pericyte death is particularly important.^4^

Hydrogen (H_2_) is a powerful antioxidant that activates the body's antioxidant system and effectively reduces O2^−^ generation.^11^ Therefore, we hypothesize that H_2_ can alleviate HI‐induced oxidative damage in pericytes caused by HI, maintain pericyte survival and improve cerebrovascular function. In 2007, Ohta first investigated the effect of H_2_ on brain injury and found that H_2_ can selectively clear cytotoxic free radicals OH and ONOO^−^, exhibiting a protective effect on acute brain ischaemia/reperfusion injury.^12^ Subsequently, H_2_ role in neurological diseases was widely promoted. To date, increasing evidence has shown protective effects of H_2_ against various neurological diseases.^13^ Moreover, at least 10 clinical trials have been conducted on the use of H_2_ in nervous system diseases, showing that H_2_ is a promising therapeutic medical gas for brain disorders.^13^ However, these studies focus mainly on adult neurological diseases, and there are relatively few studies on neonatal brain injury. Moreover, these studies mainly involve neurons, microglia and astrocytes, with almost no reports on pericytes. As pericytes role in regulating neurovascular coupling and neurological diseases becomes increasingly important, we plan to focus on exploring the improvement of pericyte biological behaviour through H_2_ therapy. This study established a hypoxic–ischaemic brain damage (HIBD) model of neonatal rats and an in vitro oxygen and glucose deprivation (OGD) model of pericytes to elucidate the effects and mechanisms of H_2_ on pericyte death, cerebrovascular function and brain function through in vivo and in vitro experiments to provide new theoretical evidence and mechanism references for the clinical application of H_2_ in HIE.

## 2 EXPERIMENTAL PROCEDURES

### 2.1 Establishment of the HIBD model in neonatal rats

All animal experiments in this study were approved by the Animal Ethics Review Committee of Sichuan University (Animal Ethics Review Pass No. WCSUH21‐2018‐034); 7‐day‐old SD rats (16–21 g, male and female) were purchased from Sichuan Dasuo Laboratory Animal Co., Ltd., China. The HIBD model was established using the Rice‐Vannucci method.^14^ Briefly, experimental rats were placed on a small‐animal special anaesthesia machine (Matrix, USA) and inhaled isoflurane. The right common carotid artery was severed with an electric coagulation knife (Aesculap, Germany). After surgical suture, the rats were recovered for 1 h, then placed in a low oxygen chamber (temperature, 37°C; 92% N_2_ and 8% O_2_) and the mixture gas was ventilated at 0.5–1 L/min for 2 h.

### 2.2 Hydrogen‐rich water (HRW) treatment

HRW (concentration: 1000 μmol/L) was purchased from Beijing Fuhydrogen Source Technology Co., China. The 7‐day‐old SD rats were randomly assigned to seven groups: normal control group: no treatment; sham group: the right common carotid artery was isolated without electrocoagulation or hypoxic ventilation; HIBD group: the right common carotid artery was isolated by electrocoagulation ionization and hypoxic ventilation; HI + HRW treatment group: after successful HIBD moulding, rats were injected intraperitoneally with HRW. The first injection was conducted immediately after moulding and the same dose of HRW was injected 24, 48 and 72 h after moulding. This group was further divided into three subgroups according to the different doses of HRW used: (1) HI + HRW‐5 (5 mL/kg), (2) HI + HRW‐10 (10 mL/kg) and (3) HI + HRW‐15 (15 mL/kg); HI + solvent group: an equal amount of distilled water was injected intraperitoneally at the time points corresponding to HRW administration. Rats in each group were killed at different time points for further examination.

### 2.3 Propidium iodide (PI) staining

PI (5 mg/kg; Sigma, USA) was injected into the tail vein of rats. After circulation for 1 h, rats were killed, their brains were removed and frozen sections were prepared. The frozen sections were baked at 37°C for 1 h, washed with PBS for 5 min and then incubated with β‐PDGFR antibody (1:100; Abcam, United Kingdom) or NeuN antibody (1:200; Abcam, United Kingdom). Finally, the sections were stained with DAPI and observed under the fluorescence microscope (Olympus, FV1000, Japan). Triple positive staining of DAPI/PI/β‐PDGFR was calculated to examine pericyte death, and triple positive staining of DAPI/PI/NeuN was calculated to examine neuronal death.

### 2.4 SOD activity

SOD activity in brain tissues was detected using an SOD detection kit (Shanghai Biyun Biotechnology Company, China).

### 2.5 Tissue immunofluorescence staining

Rat brains were dissected and embedded in an optimal cutting temperature (OCT) compound (Tissue‐Tek, Torrance, USA), and then cryosectioned to 20‐μm thickness and fixed in ice‐cold acetone. Sections were blocked with 5% normal swine serum (Vector Laboratories, Burlingame, USA) for 60 min and incubated in primary antibody overnight at 4°C. The sections were washed in PBS and incubated with Cy3‐ or Cy5‐conjugated secondary antibodies. Primary antibodies used are β‐PDGFR (1:100; Novus, USA) and ROS (1:100; Beijing Boosen Biological Company, China). Images were scanned using a digital slice scanner (3DHISTECH, Hungary) and analysed using ImageJ software.

### 2.6 Detection of brain vessel diameter and pericyte coverage

FITC‐labelled tomato lectin (Shanghai Maokang Biotechnology, China, 100 μg per rat) was injected into rats by tail vein. Rats were killed 15 min later, and the brain was prepared in frozen sections for immunofluorescence staining with β‐PDGFR antibody (1:100; Abcam, United Kingdom). The images were scanned using a digital slice scanner, the scanned images were superimposed and the vessels could be observed clearly. ImageJ software was used to select brain microvessels with a diameter <10 μm as the analysis object. The diameter was measured every 2 μm along the long axis of microvessels with a total length of 100 μm, and the average diameter was calculated. The number of β‐PDGFR‐positive pericytes covered on the microvascular per unit length (100 μm) was calculated to reflect pericyte coverage.

### 2.7 Evans blue (EB) staining

A 2% EB solution (2 mL/kg; Sigma) was injected into the rats through the tail vein. After circulation for 2 h, rats were killed and their brain tissue was homogenized in trichloroacetic acid (Shanghai Maclin Biochemical Technology company, China), and then centrifuged at 4°C, 3000 ***g*** for 20 min. The supernatant was mixed with 100% ethanol in a 1:3 ratio to prepare a mixed solvent. The mixed solvent (200 μL) was placed in a 96‐well plate, and the OD value was measured at 562 nm using a microplate reader (Molecular Devices, USA); then, EB content can be calculated from the standard curve.

### 2.8 Laser speckle imaging

The rats were anesthetized with isoflurane and their head skin was cut open to fully expose the brain. The laser beam from the RFLSI III laser speckle imaging system (Shenzhen Rayward Life Technology Company, China) was focused on the cerebral cortex 2 mm from the front fontanels and 3 mm from the middle line. Blood flow was continuously detected for 60 s and the mean CBF in the left and right cerebral hemispheres was analysed.

### 2.9 Haematoxylin and eosin (H&E) staining

Brain tissue was fixed with 4% paraformaldehyde, dehydrated with alcohol, soaked in xylene, embedded in paraffin, then cut into 5 μm slices and dried, dewaxed in xylene, treated with alcohol and stained with haematoxylin–eosin. An optical microscope (Olympus) was used to observe the pathological changes in the sections.

### 2.10 Triphenyltetrazolium chloride monohydrate (TTC) staining

The rat cerebral infarction volume was assessed using TTC staining (Sigma, USA). Each brain section was cut into four pieces and the thickness of each slice was 2 mm. The slices were then placed in a 2% TTC solution, incubated at 37°C for 20 min, the TTC solution was removed, fixed with 4% paraformaldehyde and photographed. Image‐Pro Plus software (version 6.0) was used to calculate the infarct volume. The infarct volume/brain volume ratio was used to determine the percentage of infarcts.

### 2.11 Neurofunctional score

The severity of neurological damage was evaluated using the Zea‐Longa score^15^; 0 point indicated completely normal behaviour without any neurological deficit; 1 point indicated opposite forelimb flexion, mild neurological deficit; 2 points indicated crawling opposite rotation, moderate neurological impairment; 3 points indicated standing or crawling opposite dumping, severe neurological impairment; and 4 points indicated no autonomous activity with consciousness impairment. The scoring process in this experiment was performed by three staff in double‐blind.

### 2.12 Morris water maze (MWM) test

The MWM test was conducted to evaluate the neurocognitive and motor coordination ability of rats 29 days after birth.^16^ For the first 5 days (P29‐P33), each rat was trained to swim in the four quadrants of the circular pool to detect its position navigation ability. The average escape latency time in the four quadrants was calculated and recorded as a daily final score, representing the ability to acquire spatial information. At P34, the place navigation training was conducted. The platform was removed, and the rats were released at the farthest point from the platform. The rats were allowed to swim for 60 s, and platform‐crossing times were recorded.

### 2.13 Cell culture

Rat cerebral pericytes were isolated according to the established protocols.^17^ Briefly, rat cerebral cortices were obtained from P7 SD rats. The meninges were carefully removed from the forebrains, and the grey matter was cut into 1 mm pieces in an ice‐cold Dulbecco's modified Eagle's medium (DMEM, Gibco, Rockville, USA). Homogenates were digested with collagenase type II (1 mg/mL; Sigma, St. Louis, USA) and DNase I (37.5 mg/mL; Sigma) in DMEM containing penicillin (100 U/mL) and streptomycin (100 mg/mL) at 37°C for 1.5 h with agitation. Neurons and glial cells were removed by centrifugation in 20% bovine serum albumin (BSA)‐DMEM (1000 × *g* for 20 min). Microvessels obtained from the pellets were further digested with collagenase type II (1 mg/mL) and DNase I (16.7 mg/mL) in DMEM at 37°C for 45 min with agitation. Microvessel endothelial cell clusters were separated using 33% continuous Percoll (GE Healthcare, Uppsala, Sweden) gradient centrifugation (1000 × ***g*** for 10 min). Endothelial cell clusters were pipetted and filtered through 70‐μm nylon mesh. Cell pellets were washed twice with DMEM (first at 1000 × g for 8 min, then at 700 *× g* for 5 min) and placed in uncoated culture flasks in DMEM supplemented with 10% FBS, L‐glutamine (2 mM), glucose (4.5 g/L), penicillin (100 U/mL) and streptomycin (100 mg/mL) at 37°C with a humidified atmosphere with 5% CO_2_. Cells were allowed to adhere for 4–5 h, and then non‐adherent cells were removed. After 14 days in culture, rat pericytes overgrew brain endothelial cells and reached 80%–90% confluence. Cells were used in passages 2–3 with a purity of >95% determined with NG2 and PDGFR‐β immunostaining.

### 2.14 Immunofluorescence staining for cultured pericytes

Cells on coverslips were blocked (1× PBS, 2% normal goat serum and 0.1% Triton X‐100) for 1 h and incubated overnight at 4°C with the following primary antibodies: β‐PDGFR (Abcam/Novus, USA), α‐SMA (Abcam, USA), NG2 (Abcam, USA), GFAP (Abcam, USA) and CD31 (Abcam, USA) (all diluted 1:200). Cells were washed three times with 0.1 M PBS and incubated with Cy3 or Cy5‐labelled secondary antibody (1:400; Beyotime, Shanghai, China) for 1 h at room temperature. The immunostained cells were visualized and imaged under a fluorescence microscope (Olympus, FV1000, Japan) with an excitation wavelength of 550 nm for Cy3 and 650 nm for Cy5, and an emission wavelength of 570 nm for Cy3 and 670 nm for Cy5.

### 2.15 OGD of cultured pericytes

Glucose deprivation (GD) was achieved by replacing the pericyte growth medium with glucose‐free DMEM (Gibco). Cells in GD medium were then transferred to a humidified anaerobic chamber containing 94% N_2_/5% CO_2_/1% O_2_ at 37°C. The OGD was terminated by replacing the GD medium with a normal pericyte growth medium, and the cells were returned to a normoxic incubator. Cells grown under normal conditions were used as controls.

### 2.16 HRW treatment for cultured pericytes

Immediately after OGD treatment, pericytes were treated with 1 μmol/L, 10 μmol/L or 100 μmol/L HRW for 24 h, respectively, named HRW‐1, HRW‐10 and HRW‐100 group. The control group was treated with a vehicle.

### 2.17 Cell death inhibitor treatment for cultured pericytes

To compare the effect of selected inhibitors from different cell death models with HRW, immediately after OGD treatment, pericytes were treated with 100 μM Z‐VAD. FMK (Abcam, Cambridge, MA, USA), 1 μM Fer‐1 (Sigma), 20 μM DPQ (Abcam), 30 μM Nec‐1 (Abcam), 10 mM 3‐MA (Sigma) or 10 μmol/L HRW for 24 h. The control group was treated with a vehicle. The drug dosing schedule was determined based on the results of previous reports.^18^, ^19^

### 2.18 Cell survival assay

Cell survival assays were performed using a Cell Counting Kit‐8 (CCK‐8 kit, Dojindo Co., Japan) according to the manufacturer's instructions. The luminescence was recorded using a Varioskan flash microplate reader (Thermo Fisher Scientific, Waltham, USA).

### 2.19 Oxidative injury in cultured pericytes

ROS and 8‐hydroxy‐2 deoxyguanosine (8‐OHdG) levels in cultured pericytes were determined using ROS detection kit (Shanghai BiYuntian Biotechnology Co., China) and 8‐OHdG ELISA detection kit (Wuhan Merck, Biotechnology Co., China), respectively.

### 2.20 Western blotting for cultured pericytes

Proteins were extracted from the cultured pericytes at 4°C using RIPA lysis buffer. Protein concentration was determined using the BCA Protein Assay Kit (Life, New York, USA). Proteins were resolved by SDS‐PAGE (10% polyacrylamide), transferred to a PVDF membrane and incubated with primary antibodies. The membranes were incubated with peroxidase‐labelled secondary antibodies (Life Technologies). Primary antibodies used were as follows: anti‐NRF2 antibody (1:800; MyBioSource Com), anti‐heme oxygenase 1 antibody (1:800; Gene Tex) and anti‐β‐actin (1:1000; Abcam).

### 2.21 Statistical analysis

The number of positive cells, cerebrovascular diameter and cerebrovascular peripheral cell coverage in the immunofluorescence images were calculated using ImageJ software. SPSS 22.0 software was used to process the experimental data and the data were expressed by mean ± SEM. Comparisons between two groups were performed using a *t*‐test and comparisons between multiple groups were performed using a one‐way analysis of variance (ANOVA). Statistical significance was set at *p* < 0.05. Statistical histograms were plotted using GraphPad Prism 7.0.

## 3 RESULTS

### 3.1 Effects of HRW on HI‐induced oxidative injury in pericytes in vivo and in vitro

**FIGURE 1:**
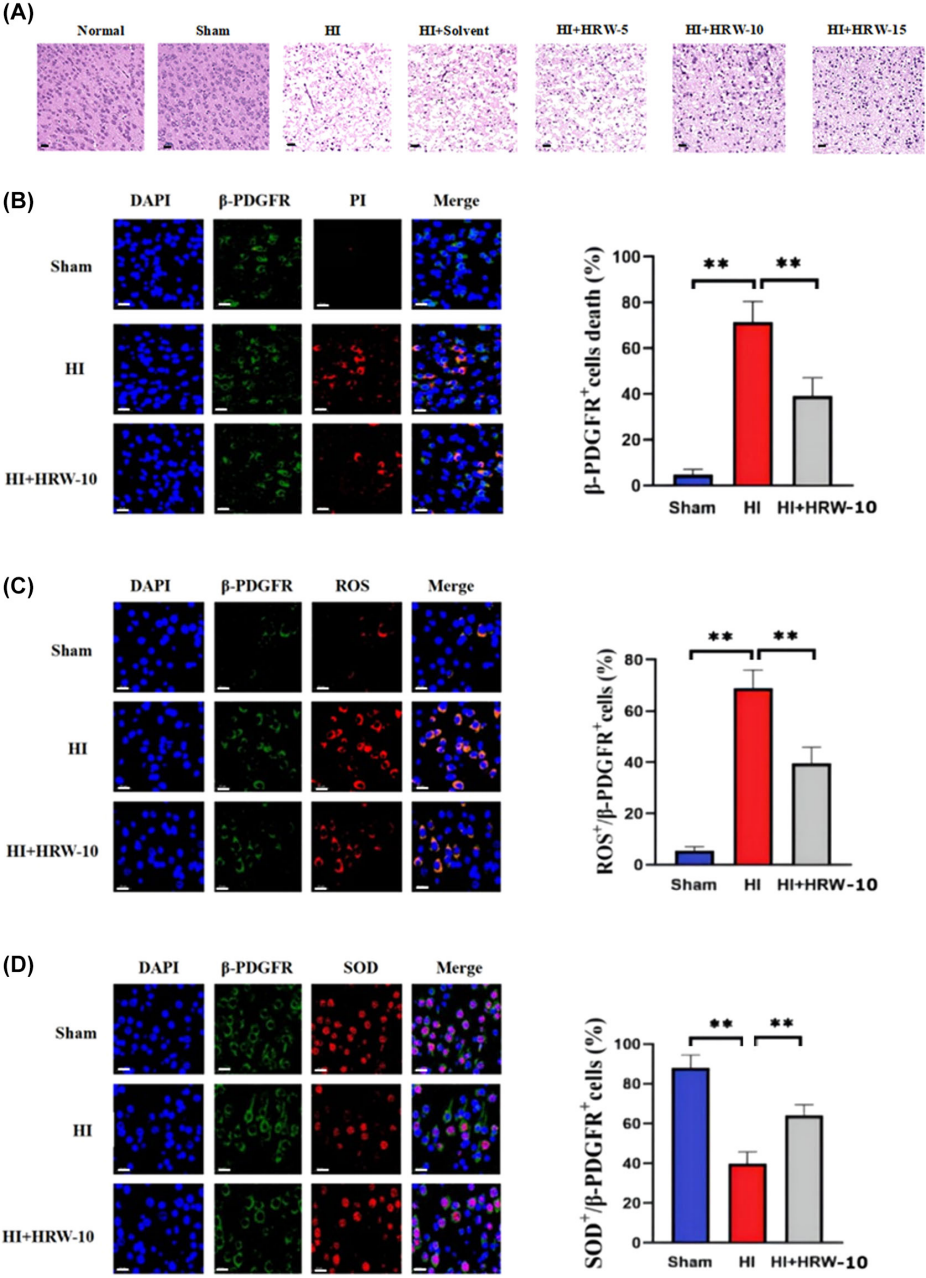
HRW alleviated brain pathological damage and oxidative injury of pericytes induced by HI in vivo. (A) H&E staining of brain tissue. (B) Triple positive staining of DAPI/PI/β‐PDGFR to show pericyte death. Data are represented as mean ± SEM (*n* = 3). (C) β‐PDGFR/ROS co‐immunostaining to show oxidative damage of pericytes. Data are represented as mean ± SEM (*n* = 3). (D) β‐PDGFR /SOD co‐immunostaining to show oxidative damage of pericytes. Data are represented as mean ± SEM (*n* = 3). HI, hypoxia‐ischaemia; HI + solvent, hypoxia‐ischemia + solvent; HI + HRW‐5, HI + HRW‐5 (5 mL/kg HRW); HI + HRW‐10, HI + HRW‐10 (10 mL/kg HRW); HI + HRW‐15, HI + HRW‐15 (15 mL/kg HRW). Scale bar = 20 μm; **p < 0.01.

**FIGURE 2:**
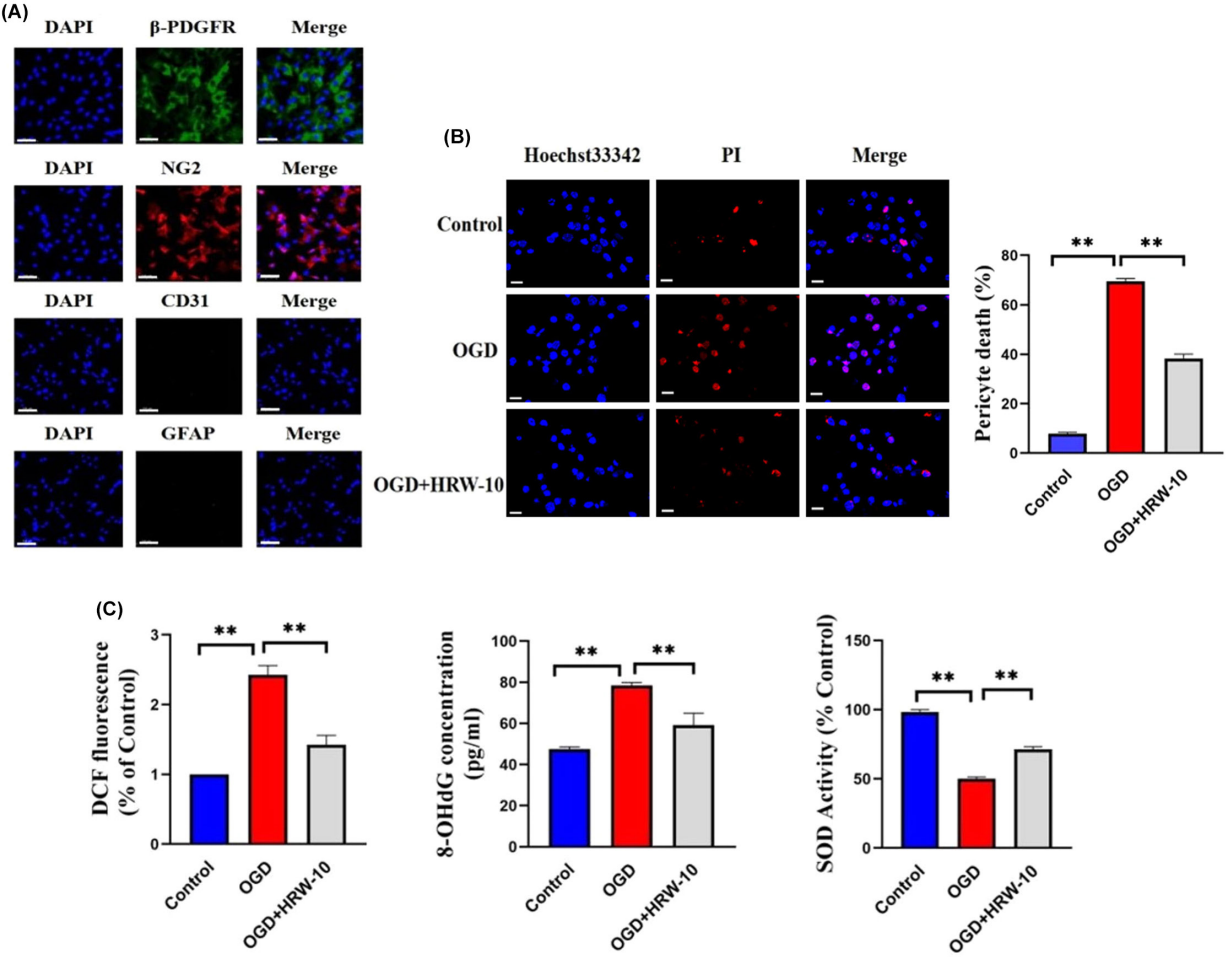
HRW alleviated the oxidative injury of pericytes induced by OGD in vitro. (A) Identification of cultured pericytes by immunostaining of cellular biomarkers. Scale bar = 20 μm. (B) Hoechst33342/PI co‐staining to show pericyte death. Data are represented as mean ± SEM (*n* = 3). Scale bar = 10 μm. (C) Oxidative damage index of pericytes. The experiment was carried out three times, *n* = 3 rats per group. Data provided as mean ± SEM. OGD, oxygen–glucose deprivation; OGD + HRW‐10, OGD + HRW‐10 (10 mL/kg HRW); **p < 0.01.

**FIGURE 3:**
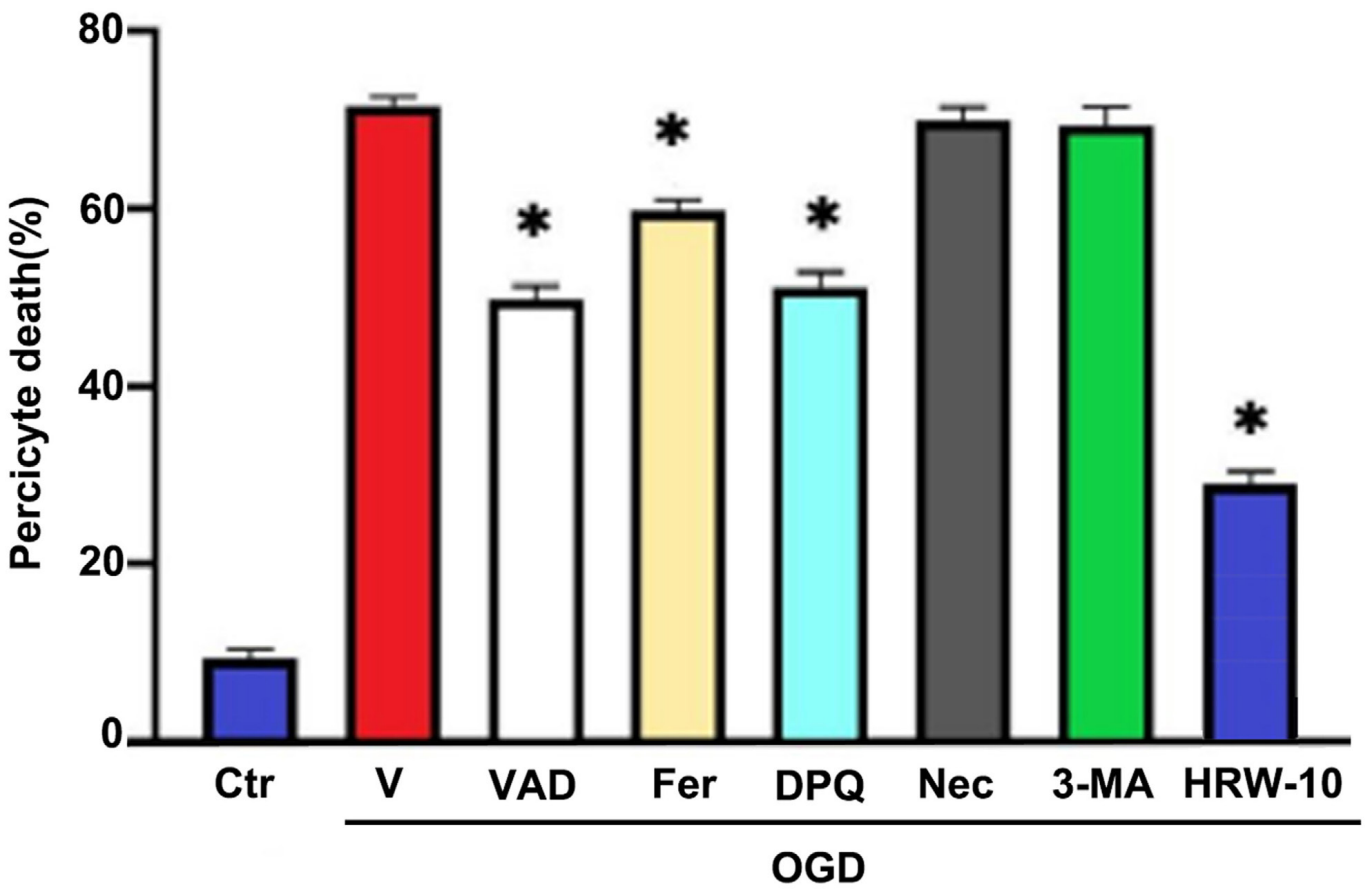
HRW is superior to a single‐cell death inhibitor and can better inhibit HI‐induced pericyte death. Data are represented as mean ± SEM (*n* = 6). Ctr, without OGD treatment; V, vehicle control; VAD, apoptosis inhibitor; Fer, ferroptosis inhibitor; DPQ, parthanatosis inhibition; Nec‐1, necroptosis inhibitor; 3‐MA, autophagy inhibitor. *p < 0.05.

H&E staining showed that after HI, the right cerebral cortex of the rats was loose, cells were disorderly arranged and some cells were severely necrotic. After HRW treatment, the pathological damage and infarct area in the brain tissue were significantly reduced. HRW‐10 was more effective than HRW‐5, and there was no significant difference between the HRW‐15 and HRW‐10 groups (Figure 1A). Therefore, we chose HRW‐10 as the treatment dose in subsequent experiments. Furthermore, there was no significant difference between the HI and HI + solvent groups (Figure 1A); therefore, we did not include the HI + solvent group in subsequent experiments. Immunostaining showed that, after HI, the number of DAPI/PI/β‐PDGFR‐positive cells in brain tissue increased, indicating obvious death of pericytes (Figure 1B). Meanwhile, the number of β‐PDGFR/ROS‐positive cells increased (Figure 1C), and the number of β‐PDGFR/SOD‐positive cells decreased (Figure 1D), showing the oxidative stress injury of pericytes after HI. HRW treatment decreased DAPI/PI/β‐PDGFR and β‐PDGFR/ROS‐positive cells while increasing the β‐PDGFR/SOD‐positive cells, indicating that HRW attenuated HI‐induced oxidative stress injury in pericytes (Figure 1B–D). This phenomenon was also observed in an in vitro study. Immunofluorescence staining was used to detect specific markers (α‐SMA and β‐PDGFR) in cultured pericytes. It showed that most cells expressed α‐SMA or β‐PDGFR, while the endothelial cell marker CD31 and the astrocyte marker GFAP were negatively stained (Figure 2A), indicating a high purity of cultured pericytes. After OGD, the survival of cultured pericytes decreased and their death increased; treatment of pericytes with HRW‐containing culture medium resulted in a significant increase in pericyte survival and a decrease in cell death, with 10 μmol/L HRW as the most effective and safe dose (Figure 2B). The SOD activity of pericytes increased, while ROS and 8‐OHdG concentrations in pericytes decreased with HRW treatment after OGD (Figure 2C). Furthermore, HRW was superior to a single‐cell death inhibitor and better inhibited HI‐induced pericyte death (Figure 3).

### 3.2 Effects of HRW on cerebrovascular function in HIBD rats

**FIGURE 4:**
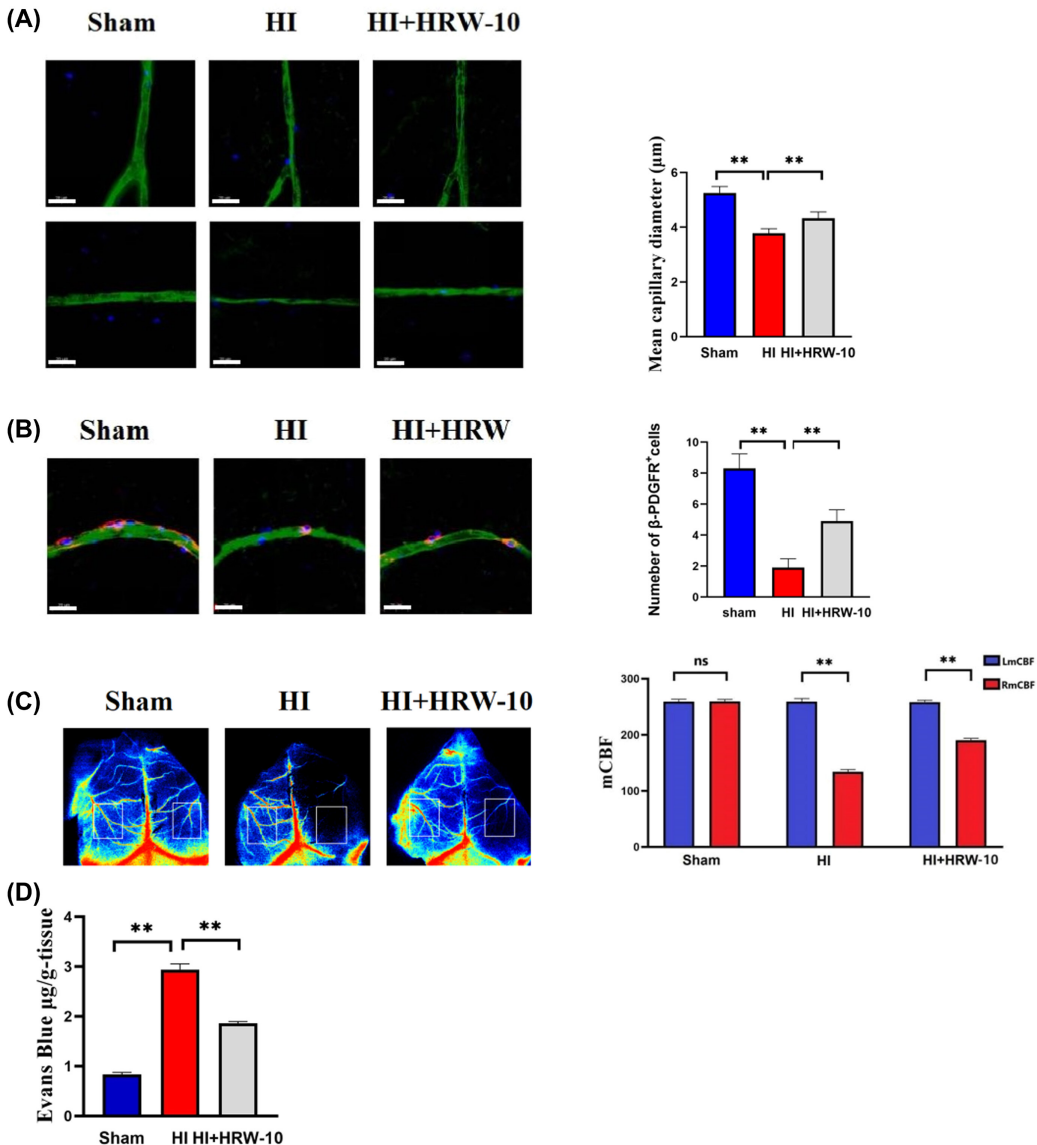
Effects of HRW on cerebrovascular function in HIBD rats. (A) Diameter of the brain blood vessels detected by lectin staining. Data are represented as mean ± SEM (*n* = 3). (B) Pericyte coverage is detected through β‐PDGFR/lectin staining. Data are presented as mean ± SEM (*n* = 3). (C) Brain CBF is detected using laser speckle imaging. Data are represented as mean ± SEM (*n* = 3). (D) BBB permeability is detected by EB dye staining. Data are represented as mean ± SEM (*n* = 3). HI + HRW, HI + HRW‐10 (10 mL/kg HRW). Scale bar = 20 μm; **p < 0.01.

Quantitative analyses of lectin staining on confocal laser scanning microscopy stacked images assessing the diameter of brain blood vessels showed that HI‐induced capillary constriction, with the diameter of brain vessels being significantly smaller in rats after HI than in sham controls, while HRW broadened the brain vessels (Figure 4A). Using dual immunostaining for β‐PDGFR and lectin, we showed a loss of pericyte coverage in HI brain compared to sham controls, while HRW increased the pericyte coverage (Figure 4B). Furthermore, the mean surface CBF on the ipsilateral side of HI rats was significantly lower than in sham controls after HI, and HRW significantly increased the ipsilateral CBF of HI rats (Figure 4C). Furthermore, extravasation of EB dye in the ipsilateral hemisphere was greater in HI rats than in sham controls, while HRW decreased BBB breakdown in the ipsilateral hemisphere of HI rats (Figure 4D).

**FIGURE 5:**
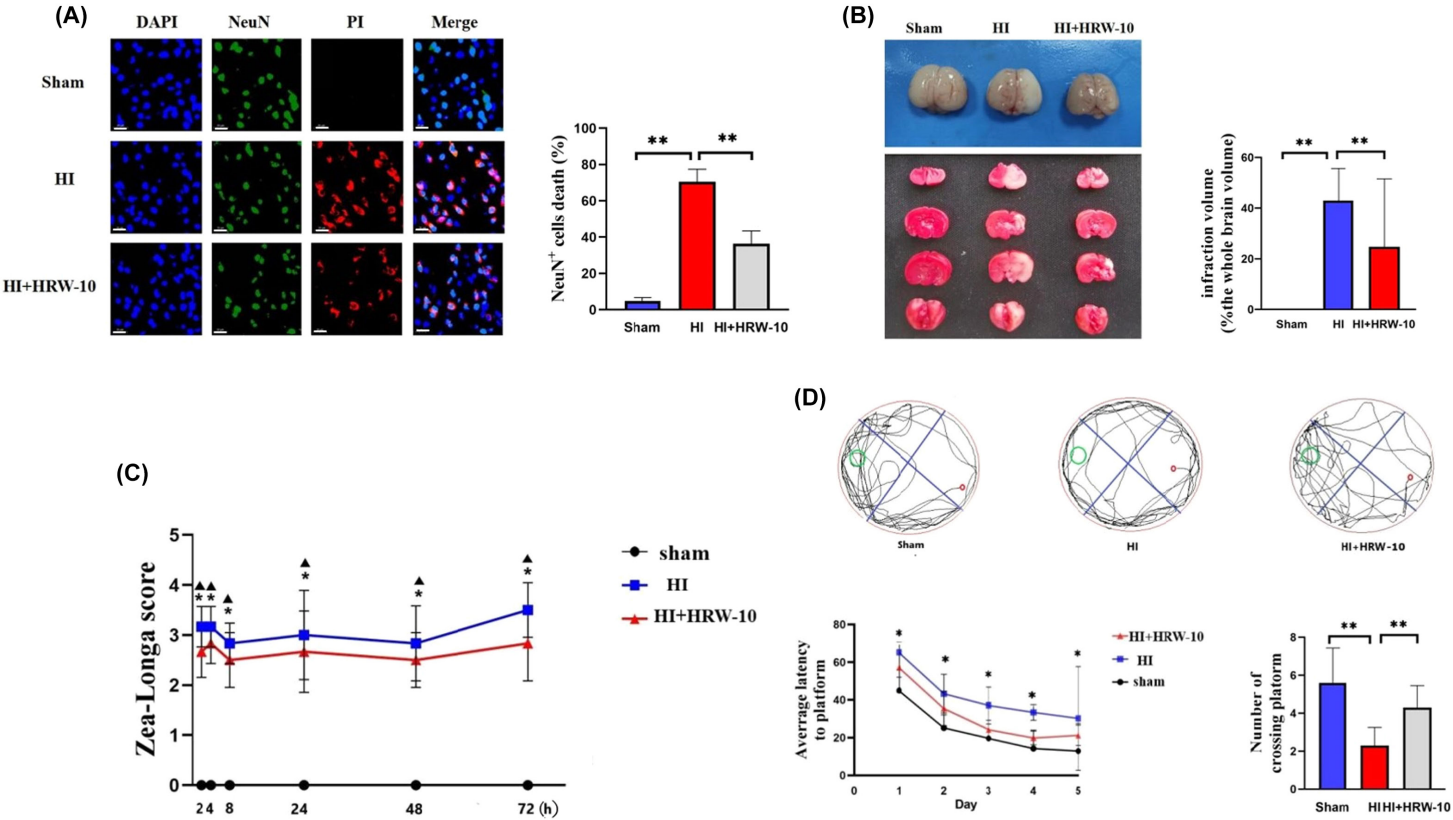
Effects of HRW on neurological function in HIBD rats. (A) Triple positive staining of DAPI/PI/NeuN to show neuronal death. Data are represented as mean ± SEM (*n* = 3). Scale bar = 20 μm. (B) Brain infarcts are detected using TTC staining. Data are represented as mean ± SEM (*n* = 3). (C) Evaluation of NSS. The experiment is carried out three times, *n* = 5 rats per group. Data provided as mean ± SEM. (D) Spatial learning and memory abilities of rats detected using the MWM. Data are represented as mean ± SEM (*n* = 9). HI + HRW, HI + HRW‐10 (10 mL/kg HRW); *p < 0.05; **p < 0.01.

Since HRW effectively restored HI‐damaged brain vessel function, we further examined its neuroprotective effects on brain function in a rat model. Triple positive staining of DAPI/PI/NeuN showed that HI induced neuronal death, while HRW significantly attenuated it (Figure 5A). TTC staining showed that HI caused severe infarcts in rat brains, while HRW decreased the infarct volume from approximately 42%–23% at 7 days after HI (Figure 5B). We determined the long‐term effects of HRW on neurological outcomes. NSS evaluation 21 days after HI showed that NSS was significantly higher in the HI group than in the sham group and decreased after HRW treatment (Figure 5C). To further verify the long‐term neurological protection of HRW in rats after HI, the MWM test was performed to evaluate spatial learning and memory 34 days after HI. The escape latency of all groups showed a downward trend in the MWM test (Figure 5D). Compared to the HI group, rats in the HRW group took significantly less time to find the underwater platform in the second quadrant on days 4 and 5 and crossed the former platform more times.

### 3.3 Liver and kidney function after HRW treatment

**FIGURE 6:**
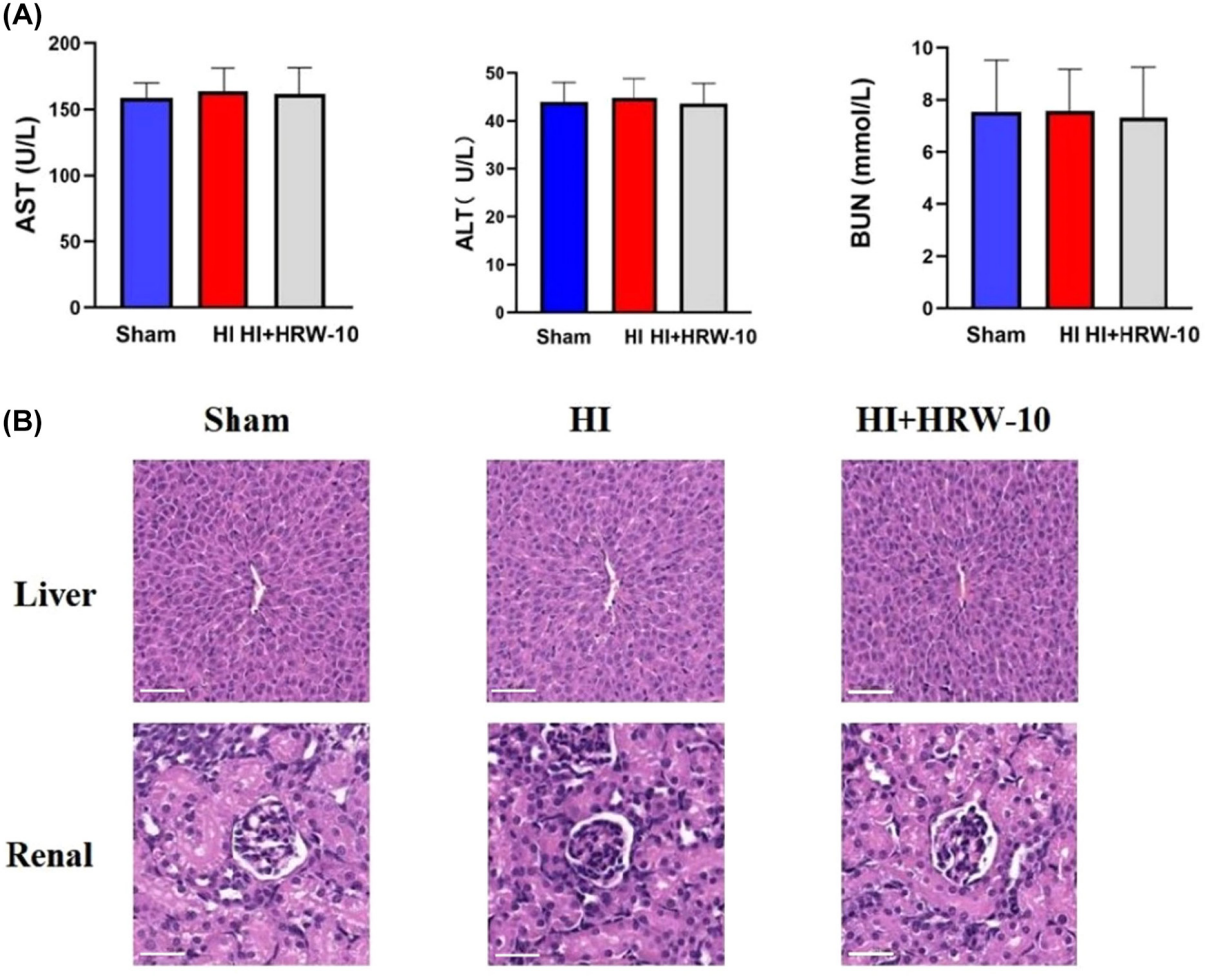
Liver and kidney function after HRW treatment. (A) Blood biochemical indicators of liver and kidney functions. Data are represented as mean ± SEM (*n* = 6). (B) Pathological changes in liver and renal tissues are detected by H&E staining. HI + HRW, HI + HRW‐10 (10 mL/kg HRW). Scale bar = 80 μm.

The liver and kidney functions (AST, ALT and BUN) of the Sham, HI and HRW groups were within the normal range (Figure 6A). Pathological changes in the liver and kidneys of the three groups were observed by H&E staining. The structures of the hepatic lobule and sinusoids were clear, the structure of the hepatic cell cord was orderly arranged and the hepatic cells were radially arranged. It also showed a normal glomerular structure without obvious proliferation of mesangial cells and mesangial matrix, no obvious inflammatory cells entering the renal interstitium and no abnormalities in the renal tubule structure (Figure 6B).

### 3.4 HRW upregulated NRF2 and heme oxygenase 1 in OGD pericytes

**FIGURE 7:**
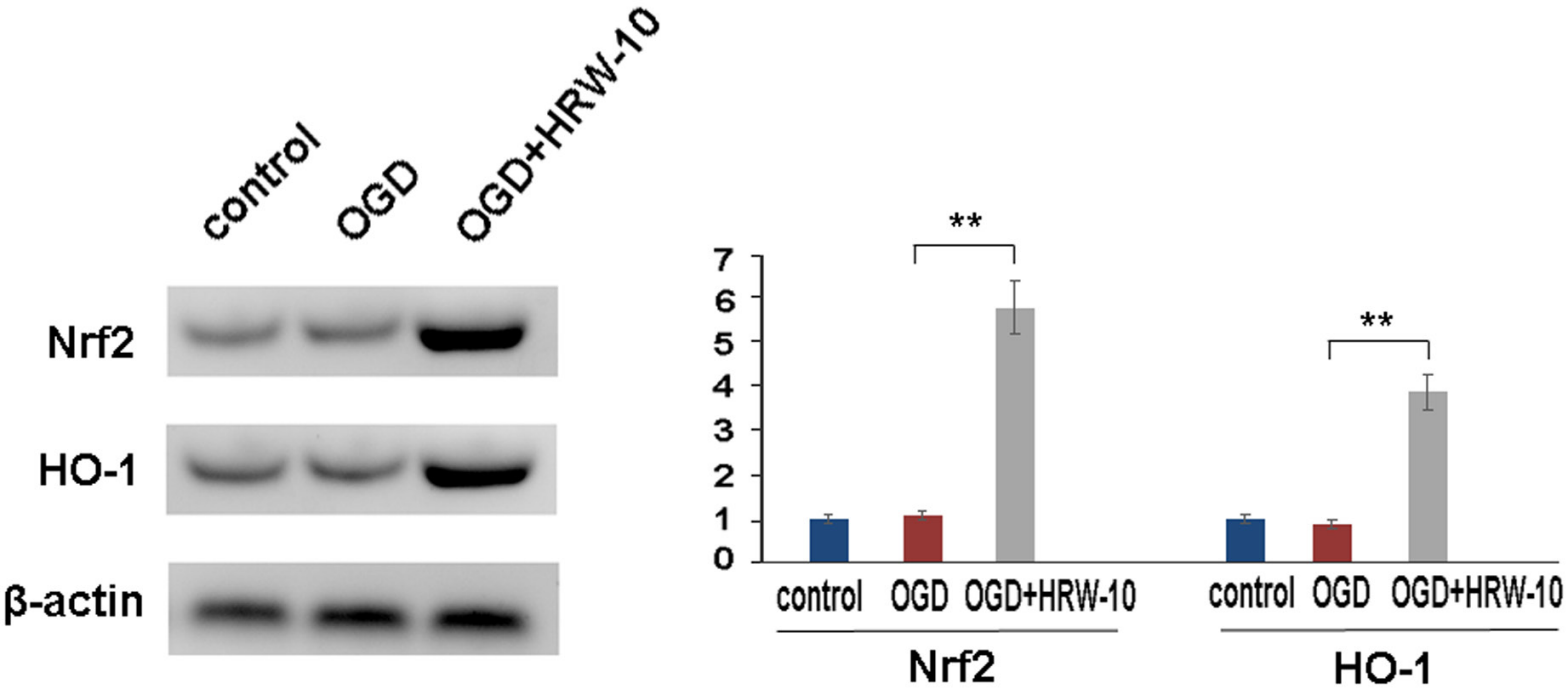
HRW upregulated NRF2 and heme oxygenase 1 in OGD pericytes. Representative western blot and densitometric analyses of NRF2 and heme oxygenase 1 are shown. Data are represented as mean ± SEM (*n* = 3). OGD, oxygen–glucose deprivation; OGD + HRW‐10, OGD + HRW‐10 (10 mL/kg HRW); **p < 0.01.

H_2_ has been known to decrease ROS through its radical scavenger and mitochondria rectifier activity.^12^, ^20^ Furthermore, H_2_ might evoke Nrf2‐HO‐1 pathway to activate antioxidant defence system,^21^, ^22^ so we detected the expression of Nrf2 and HO‐1 in OGD pericytes. Western blot showed that there was little expression of Nrf2 and HO‐1 in control pericytes. After OGD, the expression of these genes was not significantly affected, while HRW significantly upregulated their expression (Figure 7).

## 4 DISCUSSION

This study simulated the pathological damage of HIE using animal and cell models and clarified that HRW can inhibit pericyte injury and improve cerebrovascular and brain function after HI. Mechanistically, H_2_ can decrease oxidative stress in pericytes after HI, in addition to a canonical mechanism, perhaps by inducing the Nrf2‐HO‐1 pathway.

Previous reports have shown that H_2_ therapy mechanisms for brain injury include mainly antioxidant, anti‐inflammatory and anti‐apoptotic effects.^13^ These mechanisms are interconnected in a complex manner. Oxidative stress affects the expression of numerous genes, such as those related to cell apoptosis and inflammatory response; Therefore, the antioxidant effect of H2 may be its most fundamental property.^23^ To date, there are two main hypotheses on the antioxidant mechanism of H_2_. The widely accepted hypothesis is that H_2_ reacts directly with OH and ONOO^−^, which is the traditional ‘scavenger theory’.^12^ Another view is that H_2_ inhibits ROS production by acting as a rectifier for mitochondrial electron flow.^20^ In addition to its properties as a free radical scavenger and rectifier of the mitochondrial respiratory chain, H_2_ treatment might induce adaptive responses to oxidative stress by evoking the Nrf2 antioxidant defence system.^21^, ^22^ Nrf2 is a positive regulator of the human antioxidant response element, modulating the expression of hundreds of genes, most of which are antioxidant genes, including HO‐1 and SOD.^24^ It has been reported that a relevant source of ROS stems from mitochondrial complex I inhibition; HO‐1 upregulation can protect against complex I inhibitors and decrease ROS production.^25^, ^26^ Therefore, HO‐1 is considered an important anti‐oxygen stress enzyme that plays a role in many types of tissue injury. Based on this, we examined Nrf2 and HO‐1 levels using western blotting. It showed that HRW treatment significantly increased Nrf2 and HO‐1 expression in pericytes, suggesting that, besides the canonical mechanism, the Nrf2‐HO‐1 pathway is another HRW mechanism to modulate ROS production and subsequent oxidative stress injury.

HI can induce various forms of cell death.^27^ To compare the effect of different death inhibitors and HRW on pericyte death after HI, we tested OGD‐induced cell death after treatment with inhibitors of different death modes. The results showed that the death patterns of brain pericytes induced by HI include apoptosis, parthanatosis and ferroptosis; however, by inhibiting a single specific death mode, pericytes death cannot be fully suppressed. Previously, oxidative stress was shown to be the cause of multiple modes of cell death.^28^ When subjected to various harmful stimuli, excessive ROS and reactive nitrogen species are produced in cells.^29^ Excess ROS and reactive nitrogen species can lead to lipid peroxidation and oxidative damage to proteins and DNA. Membrane lipid peroxidation not only leads to changes in membrane function but also to structural damage, ultimately leading to cell rupture. Membrane lipid peroxidation is involved in various types of programmed cell death, such as ferroptosis,^30^ necroptosis,^31^ parthanatos,^32^ autophagy,^33^ etc. Oxidative DNA damage is closely related to the occurrence of apoptosis and parthanatos.^34^, ^35^ Given that HI‐induced activation of apoptosis, parthanatosis and ferroptosis is closely related to the earlier response to oxidative stress in cells, reducing oxidative stress of pericytes is an effective strategy to inhibit pericyte damage. In this study, we found that HRW was superior to a single‐cell death inhibitor and better inhibited HI‐induced pericyte death, confirming this conjecture.

Hydrogen has several advantages as a neuroprotective gas. It can cross the BBB, penetrate the cell membrane and diffuse into the cytosol and organelles.^13^ Furthermore, repeated administration of H_2_ does not cause tolerance.^36^ There are multiple routes of H_2_ administration, including inhalation of H_2_ gas, drinking HRW, injection of H_2_‐rich saline (HRS), HRW bathing, intake of a solid H_2_ carrier (coral calcium hydride) and H_2_‐producing precursors, as well as functional micro/nanomaterials for targeted H_2_ delivery.^13^ Each method of administration has its advantages and disadvantages.^37^, ^38^ As 7‐day‐old newborn rats cannot drink water spontaneously, in this experiment, HRW is administered intraperitoneally. Considering that babies born with severe asphyxia usually require assisted ventilation, inhalation of H_2_ gas could be a direct and effective way of H_2_ administration for babies with neonatal HIE.^39^ Although many studies have shown that H_2_ has no toxic effects, adverse events, such as diarrhoea and heartburn have been reported in individual cases.^13^ Fortunately, this study showed that the liver and kidney functions of rats were unaffected when using the present dose of HRW.

Several studies have shown the neuroprotective effect of H_2_ on neonatal HIBD. Intraperitoneal injection of HRS significantly promotes M2 microglia polarization and suppresses neuro‐inflammation, further restoring behavioural deficits in a neonatal mouse model of HI.^40^ Similar results have been obtained from HI models in neonatal rats and piglets.^41^, ^42^ In a rat model of neonatal HIBD, H_2_ inhalation has been shown to inhibit neuronal loss and astrocyte activation, further reducing the infarct size of the brain.^41^ Another study in a 5‐day neonatal hypoxia/ischaemia piglet model showed that H_2_ ventilation combined with mild hypothermia improves the neurological score and improves the motor function of piglets.^42^ A recent study showed that H_2_ combined with therapeutic hypothermia ameliorated seizure burden after HI insult in newborn piglets.^43^ These studies mainly involve the effects of H_2_ on neurons, microglia and astrocytes. There have also been studies attempting to explore H_2_ gas role from the perspective of regulating neurovascular reactivity,^44^, ^45^ but no in‐depth research has been conducted on its mechanism. In recent years, neurovascular coupling role in physiological function and pathological damage to the nervous system has been increasingly valued.^46^ Recent studies have found that pericytes are a key factor in the neurovascular coupling system,^47^ prompting researchers to further explore pericytes role in neurological diseases. Our study elucidates the role and mechanism of H_2_ in treating HIBD from the perspective of pericytes, providing new theoretical evidence and mechanism references for the clinical application of H_2_ in HIE.

## AUTHOR CONTRIBUTIONS

**Hui Li:** Writing – original draft (equal). **Hao Sun:** Writing – original draft (equal). **Shiping Li:** Data curation (equal). **Lingyi Huang:** Data curation (equal); methodology (equal). **Mingfu Zhang:** Data curation (equal). **Shaopu Wang:** Data curation (equal); methodology (equal). **Qian Liu:** Data curation (equal). **Junjie Ying:** Data curation (equal). **Fengyan Zhao:** Data curation (equal). **Xiaojuan Su:** Data curation (equal). **Dezhi Mu:** Writing – review and editing (equal). **Yi Qu:** Conceptualization (equal); writing – original draft (equal); writing – review and editing (equal).

## FUNDING INFORMATION

This work was supported by the National Natural Science Foundation of China (82371717, 82271749, 82201905 and 82241036); National Key R&D Program of China (2021YFC2701700 and 2021YFC2701704); and Grant from the Science and Technology Bureau of Sichuan Province of China (2023NSFSC0544, 2024NSFSC0047).

## CONFLICT OF INTEREST STATEMENT

The authors declare that they have no competing interests.

## Data Availability

The data that support the findings of this study are available on request from the corresponding author.
